# Estimation of dietary ^14^C dose coefficient using ^13^C-labelled compound administration analysis

**DOI:** 10.1038/s41598-020-64954-w

**Published:** 2020-05-18

**Authors:** Tsuyoshi Masuda, Toshitada Yoshioka, Tomoyuki Takahashi, Hiroshi Takeda, Hideo Hatta, Kensaku Matsushita, Yasuhiro Tako, Yuichi Takaku, Shun’ichi Hisamatsu

**Affiliations:** 1 Institute for Environmental Sciences, Aomori, Japan; 2 Hirosaki Gakuin University, Aomori, Japan; 3 Kyoto University, Osaka, Japan; 4 National Institute of Radiological Sciences, Chiba, Japan; 5 University of Tokyo, Tokyo, Japan

**Keywords:** Metabolism, Nuclear energy

## Abstract

Carbon-14 released from nuclear facilities has been assessed to contribute significantly to the radiation dose that people are exposed to through the food chain. However, the current dose coefficient for members of public, which is the ratio of the 50-year committed effective dose to ingested 1 Bq ^14^C, recommended by the International Commission on Radiological Protection (ICRP) is not based on experimental human metabolic data for ^14^C in nutrients and diet. Therefore, to validate the coefficient, we administered ^13^C-labelled nutrients consisting of four amino acids, three fatty acids, and one monosaccharide to volunteers as substitutes for ^14^C labelled nutrients and measured the ^13^C concentration in various excreta samples. Although metabolic models were constructed from the excretion data, a significant fraction of administered ^13^C was not recovered from some nutrients. The dose coefficients of ^14^C in uniformly labelled Japanese diet, which were estimated under several assumptions about the unrecoverable fraction, varied from (6.2 ± 0.9) × 10^–11^ to (8.9 ± 4.4) × 10^–10^ Sv Bq^−1^ and were approximately comparable to the current value of 5.8 × 10^–10^ Sv Bq^−1^ recommended by the ICRP. Further studies are necessary to elucidate the metabolism of ^14^C in various nutrients in the unrecoverable fraction.

## Introduction

Carbon-14 is released from places such as nuclear fuel reprocessing facilities and nuclear power plants^1^. It is a major radionuclide contributing to the radiation dose to people around the nuclear fuel reprocessing facilities^1–3^. The dose coefficient of ^14^C, the ratio of 50-year committed effective dose to ingested 1 Bq ^14^C, is an important parameter for the dose evaluation. The International Commission on Radiological Protection (ICRP), which provides recommendations and guidance on radiological protection, has developed models describing the biokinetics of radionuclides for the calculation of doses from data of intakes or bioassay samples. For estimating the dose coefficient of ^14^C in diet of members of public, the ICRP has utilized a generic one-compartment model for organic carbon metabolism^4^, whereas a multi-compartment model was used in a recent publication for unspecified ^14^C compounds ingested by workers^5^. The one-compartment model for members of public assumes that ^14^C is uniformly distributed throughout all body organs and tissues following entry into the systemic circulation. The organic-carbon compartment of the model has a half-life of 40 days, which is derived from the mass balance of carbon in the reference man of 70 kg body weight with body carbon weight of 16 kg and carbon intake and excretion of 300 g carbon per day each^6^. The dose coefficient of organic ^14^C in the diet (5.8 × 10^−10^ Sv Bq^−1^) was defined using that metabolic model^7^, which is not based on actual experimental data obtained from ^14^C in foods or component compounds of major nutrients. Therefore, experimental data of ^14^C in those compounds are needed to validate the generic one-compartment model of the ICRP or revise it in future publications for members of public following publications for workers^8^.

In rat tissue and organs, some studies have examined the biokinetics of ^14^C incorporated into foods or component molecule of major nutrient, e.g., single oral administration of [^14^C(U)]-glucose, [^14^C(U)]-glycine, [^14^C(U)]-leucine, [^14^C(U)]-lysine, [1–^14^C]-oleic acid, [1-^14^C]palmitic acid, and [2-^14^C]-thymidine^9^; and continuous ingestion of ^14^C-wheat^10^, [^14^C(U)]-glycine, [^14^C(U)]-leucine, and ^14^C-rapeseed^11^. In sheep, Crout *et al*. reported the biokinetics of ^14^C after oral administration of ^14^C-glucose^12^. However, most of the human data has been restricted to the ^14^C in drug molecules in biomedical studies^13^ and diagnostic use such as ^14^C-glycocholic acid and ^14^C-xylose breath tests^14^, and only few investigations have reported the biokinetics of ^14^C in component molecule of major nutrients. Malmendier *et al*.^15^ examined the metabolism of ^14^C in intravenously administered [1-^14^C]-glucose, [1-^14^C]-glycerol, and [1-^14^C]-palmitate for 24 h in breath in normal and hyperlipemic patients. Simmons *et al*.^16^ reported the ^14^C excretion in urine and breath until 3 and 7 days, respectively, after intravenous [1-^14^C]-alanine administration for human volunteers. However, both studies monitored the breath for a short time of up to 7 days after the administration. Berlin and Tolbert^17^ reported the breath excretion of ^14^C following intravenous injection of glycine-[2-^14^C] decreased in four successive phases with half-lives of 0.12, 0.97, 6.1, and 71.5 days. Stenström *et al*.^18^ orally administered ^14^C-triolein and found two phase excretions in the breath with half-lives of 2 and several hundred days. Gunnarsson *et al*.^19^ also reported that the excretion of ^14^C in breath after oral ^14^C-triolein administration decreased in three phases with half-lives of 0.04, 2, and 150 days. Although the data are limited, several studies have reported fractions of excreta with longer half-lives than that of the ICRP generic one-compartment model as described above.

Ingested carbon is excreted via several physiological routes including in the breath, urine, faeces, hair, nails, epithelial cells of the skin and mucous membrane, mucus, and other secretions. The ICRP has classified the routes into four excretion pathways, breath, urine, faeces, and others (other*, hereafter) and estimated the distribution ratio of carbon via the pathways in their reference man^6^. However, the previous studies on carbon excretion in humans described above examined only the respiration and urinary pathways. Regarding the other* pathway, Stenström *et al*. reported the specific activity of ^14^C in hair from personnel at a research laboratory and pointed out the possibility of using hair as a bioassay sample^20^. To the best of our knowledge, no data has been reported for the human body on the metabolism of ^14^C incorporated into compounds covering a broad range of nutrients and food, considering every four excretion routes of the ICRP.

We previously reported the biokinetics of carbon in the human body estimated using data of the ^13^C/^12^C ratio in breath, urine, faeces, and hair, representing carbon excretion through the breath, urine, faeces, and other* pathways, respectively, after a single oral administration of ^13^C-labelled glucose to three healthy male volunteers^21^. The ^13^C/^12^C ratio in breath, urine, and hair samples was measured for up to ~112 days after administration. The excretion of ^13^C in the evaluated pathways decreased in two exponential phases with fast and slow rates, and the ^13^C/^12^C ratio of the other* pathway was higher than that of the breath and urine pathways toward the end of the experiment. Although this finding suggests that the carbon pool of the other* pathway had a longer half-life than that of the breath and urine pathways, we could not get the statistically significant ^13^C half-life of the slow decreasing fraction in the other* pathway.

In this paper, we report the results of experiments administering ^13^C-labelled four amino acids and three fatty acids to volunteers. First, ^13^C-labelled leucine and palmitic acid were orally administered once to the volunteers and the excretion through the four routes of interest were measured for up to ~160 day after the administration. Then, the contribution of carbon pools of each route to the cumulative burden of ^14^C for 50 years was calculated assuming the same metabolism of ^13^C and ^14^C. Since the urine and faeces routes did not have significant contributions, we focused on only the breath and other* routes in experiments on the remaining nutrient components. In addition, once daily dosing administered four successive times, instead of the previously used single administration, was adopted for a more accurate measurement of ^13^C excretion in the final stage of the experiments. An experiment on ^13^C-labled glucose administered four times was also conducted and, finally, the dose coefficient of ^14^C in the Japanese diet was estimated using integrated biokinetic models of each nutrient component.

## Results and Discussion

### Design of experiments

We selected the following amino acids as representative of proteins: leucine from branched chain amino acids, glutamic acid from major amino acids, glycine from collagen components, and phenylalanine from aromatic amino acids. Palmitic, oleic, and linoleic acids were selected as representative saturated, mono-unsaturated, and poly-unsaturated fatty acids, respectively while carbohydrates were represented by glucose. All carbon atoms of the compounds used were labelled with ^13^C (Cambridge Isotopes, Inc., Tewksbury). The compounds were administered to healthy volunteers and samples of various excreta were collected from them and analysed for ^13^C. All the ^13^C/^12^C ratio data presented in this report are shown as increments from the background values measured for the samples before administration, and normalized to the same dose per body weight (1 g ^13^C/70 kg body weight) prior to the following analysis. When the ^13^C/^12^C ratio under the detection limits, <95% confidence limit of the background, was observed, the data of the detection day and after it were not used for the subsequent numerical analysis. The detection limit mostly depended on the amount of ^13^C administered per body weight and individual difference.

### Selection of major excretion routes of ^13^C in amino acid and fatty acid

**Figure 1:**
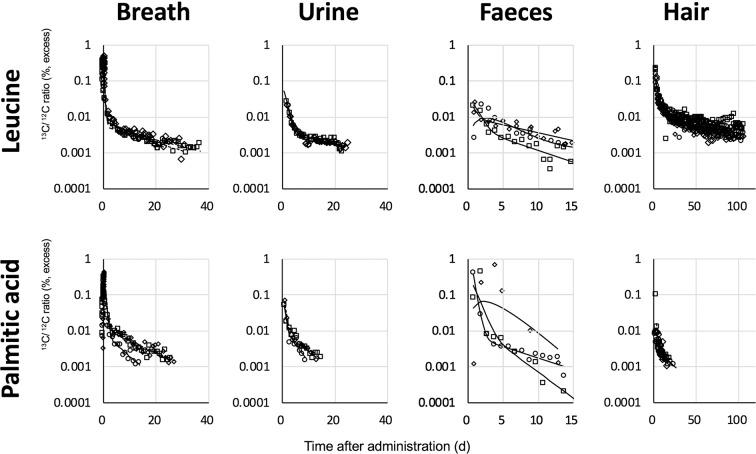
Normalized ratio of ^13^C/^12^C in excreta (breath, urine, faeces, hair) after oral administration of ^13^C-leucine and ^13^C-palmitic acid. Circles, diamonds, and squares represent volunteer 1, 2, and 3, respectively. Lines were fitted using model developed in this study.

We orally administered ^13^C-labelled leucine or palmitic acid to three male volunteers who were sedentary workers and then collected their breath, urine, faeces, and hair samples at different times depending on the compounds and excreta (Fig. 1). As shown in Figure 1, the ^13^C/^12^C ratio of the breath and hair samples increased after administration then decreased in two exponential phases. The peak time of breath samples from the leucine-administered group (0.11 ± 0.02 days, mean ± standard deviation) was earlier than that of the palmitic acid-administered group (0.30 ± 0.03 days, P < 0.01), suggesting that the compounds had different absorption rates from the alimentary tract (Fig. S1 in the supplementary information). The peak time of the ^13^C/^12^C ratio in faeces samples after administration of labelled leucine and palmitic acid varied from 0.75 to 1.9 days and from 0.75 to 3.75 days among subjects, respectively. The excretion rate of administered ^13^C into the faeces is considered to depend on subject-specific values such as residence time of faeces in the colon, which was independent of the administered compounds.

**Figure 2:**
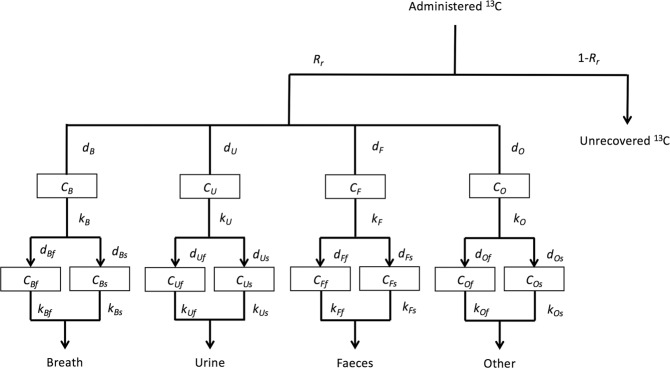
Structure of model developed to analyse ^13^C excreted through each excretion pathway after ingestion of ^13^C labelled compounds. *R*_*r*_ is ratio of total recoverable carbon through four excretion pathways to administered ^13^C. *d*_*m*_*, d*_*mn*_, distribution factors of ^13^C to each compartment. *C*_*m*_*, C*_*mn*_, ^13^C mass in the carbon pool (g). *k*_*mn*_, excretion rate constant (day^−1^).

For each compound, we developed a metabolic model comprising four carbon excretion pathways: breath, urine, faeces, and other* (Fig. 2). Since the ^13^C/^12^C ratios of each excreta sample showed a dual exponential decrease after peaking, two compartments representing fast and slow carbon metabolism were set for each of the four excretion pathways. However, every compartment in those models does not represent any tissue and organ. In addition, one upstream compartment was used for each pathway to fit the prediction curve of the model to the increase phase observed in the breath and faeces samples immediately after administration (Fig. 1 and Fig. S1).

The rate of ^13^C excretion for each pathway (*E*_*m*_, g d^−1^) is given by the following equations:

1 $${E}_{m}=\sum _{n}{k}_{mn}\frac{{d}_{m}{d}_{mn}{k}_{m}}{{k}_{m}-{k}_{mn}}({e}^{-{k}_{mn}t}-{e}^{-{k}_{m}t})$$

where, *m* is the excretion pathway symbol of *B, U, F*, and *O* corresponding to the excretion pathways of breath, urine, faeces, and other*, respectively; *n* is the symbol of *f* and *s* corresponding to the compartments with fast and slow metabolism, respectively; *k*_*m*_ and *k*_*mn*_ are the excretion rate constants from compartment *C*_*m*_ and *C*_*mn*_, respectively; *d*_*m*_ is the distribution ratio of ^13^C into compartment *C*_*m*_ to the administered ^13^C; *d*_*mn*_ is the distribution ratio of ^13^C into compartment *C*_*mn*_ to ^13^C from compartment *C*_*m*_; *R*_*r*_ is the recovery ratio of administered ^13^C. The sum of distribution ratios of compartments with fast and slow metabolism was fixed to unity (i.e. *d*_*Bf*_ + *d*_*Bs*_ = 1). Since the sum of the distribution ratios of each pathway (*d*_*B*_ + *d*_*U*_ + *d*_*F*_ + *d*_*O*_) was not forced to unity, to prevent underestimation of these parameters, *R*_*r*_ was over unity for some cases.

The parameter values were estimated using the data of each volunteer using a least square method (Table 1). Although some parameters were not statistically significant (p > 0.05, most parameters for the urine, faeces, and other* pathway in the palmitic acid model, and in the faeces pathway in the leucine model), we included those parameters in the following analysis.

**Table 1:** Parameters are explained in Fig. 2.

Parameter	Volunteer										
	No. 1			No. 2			No. 3			All	
	Value	S.E.	P-value	Value	S.E.	P-value	Value	S.E.	P-value	Mean	S.D.
**(A) Estimated parameters of developed model for** ^13^C in leucine.											
*d*_*B*_	0.62	0.02	<0.01	0.62	0.02	<0.01	0.76	0.01	<0.01	0.68	0.05
*d*_*Bf*_	0.50	0.02	<0.01	0.45	0.02	<0.01	0.52	0.02	<0.01	0.48	0.02
*d*_*U*_	0.012	0.002	<0.01	0.011	0.003	<0.01	0.011	0.003	<0.01	0.011	0.001
*d*_*Uf*_	0.46	0.12	<0.01	0.53	0.12	<0.01	0.35	0.13	<0.01	0.46	0.09
*d*_*O*_	0.24	0.01	<0.01	0.36	0.02	<0.01	0.22	0.01	<0.01	0.30	0.12
*d*_*Of*_	0.40	0.02	<0.01	0.34	0.03	<0.01	0.38	0.03	<0.01	0.40	0.03
*d*_*F*_	0.014	0.431	0.97	0.0083	0.0028	<0.01	0.0047	0.0004	<0.01	0.0092	0.0049
*d*_*Ff*_	0.28	4.0e+01	0.99	0.13	1.2e+02	>0.99	0.51	2.8e+00	0.86	0.30	0.17
*k*_*B*_ (d^−1^)	5.2	0.7	<0.01	6.9	1.5	<0.01	4.2	0.6	<0.01	5.6	0.5
*k*_*Bf*_ (d^−1^)	20	5	<0.01	16	5	<0.01	17	4	<0.01	18	3
*k*_*Bs*_ (d^−1^)	0.076	0.009	<0.01	0.064	0.007	<0.01	0.043	0.004	<0.01	0.061	0.015
*k*_*Uf*_ (d^−1^)	0.72	0.17	<0.01	0.96	0.30	<0.01	0.70	0.19	<0.01	0.82	0.18
*k*_*Us*_ (d^−1^)	0.024	0.012	0.05	0.042	0.016	<0.01	0.021	0.014	0.15	0.030	0.010
*k*_*Of*_ (d^−1^)	0.29	0.03	<0.01	0.35	0.05	<0.01	0.28	0.04	<0.01	0.32	0.07
*k*_*Os*_ (d^−1^)	0.013	0.001	<0.01	0.012	0.001	<0.01	0.014	0.001	<0.01	0.012	0.002
*k*_*F*_ (d^−1^)	0.43	1.1e+03	>0.99	2.9	1.1e+04	>0.99	1.7	9.3e+01	0.99	1.7	1.2
*k*_*Ff*_ (d^−1^)	0.43	1.1e+03	>0.99	2.8	1.1e+04	>0.99	1.5	0.0	<0.01	1.7	1.2
*k*_*Fs*_ (d^−1^)	0.0099	4.7e-01	0.98	0.10	0.02	<0.01	0.17	9.6e+01	0.99	0.087	0.072
*R*_*r*_	0.89			1.2			0.91			1.0	0.2
**Parameter**	**Volunteers**										
	**No. 4**			**No. 5**			**No. 6**			**All**	
	**Value**	**S.E**.	**P-value**	**Value**	**S.E**.	**P-value**	**Value**	**S.E**.	**P-value**	**Mean**	**S.D**.
**(B) Estimated parameters of developed model for** ^**13**^**C in palmitic acid**											
*d*_*B*_	0.62	0.08	<0.01	0.92	0.07	<0.01	0.77	0.07	<0.01	0.80	0.21
*d*_*Bf*_	0.73	0.30	0.02	0.53	0.15	<0.01	0.46	0.06	<0.01	0.59	0.14
*d*_*U*_	0.0062	0.0017	<0.01	0.0088	0.0050	0.08	0.0080	0.0014	<0.01	0.0077	0.0013
*d*_*Uf*_	0.52	0.85	0.05	0.55	0.22	0.01	0.40	0.12	<0.01	0.49	0.08
*d*_*O*_	0.017	0.021	0.04	0.040	0.071	0.57	0.051	6.3	0.99	0.036	0.017
*d*_*Of*_	0.21	0.63	0.07	0.66	0.61	0.28	0.23	2.8e+01	0.99	0.37	0.26
*d*_*F*_	0.057	0.430	0.09	0.030	0.012	0.01	0.024	0.006	<0.01	0.037	0.018
*d*_*Ff*_	0.94	0.49	0.06	0.75	1.3e+07	>0.99	0.84	1.2e+01	0.94	0.84	0.10
*k*_*B*_ (d^−1^)	2.8	5.6e+01	0.96	2.7	2.0e+01	0.89	4.2	2.3	0.07	3.1	0.93
*k*_*Bf*_ (d^−1^)	2.8	5.6e+01	0.96	2.7	2.0e+01	0.89	1.8	0.8	0.02	2.4	0.5
*k*_*Bs*_ (d^−1^)	0.15	0.04	<0.01	0.076	0.024	<0.01	0.081	0.012	<0.01	0.10	0.04
*k*_*Uf*_ (d^−1^)	2.8	5.7e+01	0.96	1.8	8.8	0.83	1.4	0.8	0.09	2.1	0.7
*k*_*Us*_ (d^−1^)	0.22	0.09	0.02^‡^	0.12	0.12	0.29	0.14	0.03	<0.01	0.16	0.05
*k*_*Of*_ (d^−1^)	0.60	1.3e+00	0.64	0.71	0.65	0.27	0.23	0.31	0.45	0.52	0.26
*k*_*Os*_ (d^−1^)	0.051	0.040	0.21	0.045	1.1e-01	0.68	0.0044	7.5e-01	0.99	0.033	0.025
*k*_*F*_ (d^−1^)	2.7	2.0	0.18	0.45	1.3e+03	>0.99	1.9	6.5e+02	>0.99	1.7	1.1
*k*_*Ff*_ (d^−1^)	8.2	1.7e+02	0.96	0.45	1.5e+03	>0.99	1.9	6.5e+02	>0.99	3.5	4.1
*k*_*Fs*_ (d^−1^)	0.15	0.04	<0.01	0.45	4.1e+03	>0.99	1.9	0.1	<0.01	0.31	0.15
*R*_*r*_	0.70			1.1			0.85			0.89	0.21

S.E., Standard error. S.D., Standard deviation. *Rr*, recovery ratio (i.e. *dB* + *dU* + *dF* + *dO*).

**Figure 3:**
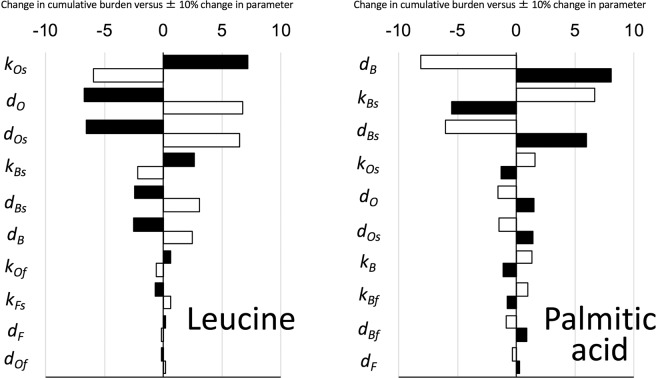
Sensitivity analysis of model parameters. A, leucine and B, palmitic acid. Changes in 50-year cumulative burden versus ±10% changes in parameter values were calculated. The top 10 parameters are shown in decreasing order. Solid and open bars, −10% and +10% change in parameters, respectively.

We calculated the 50-year cumulative body burdens of ^13^C from orally ingested 1 g ^13^C in leucine and palmitic acid using the above models. Then, the variation of the burden in response to a ± 10% change of each parameter was calculated using the mixed data from all subjects for each compound group. The results are shown in Fig. 3 for the top 10 parameters causing larger variation and most were in the breath and other* pathways, whereas only two parameters causing smaller variations in the faeces pathway were included. All the statistically insignificant parameters in the regression analysis were not sensitive to the burden. The contribution of urine and faeces pathways to the burden from orally ingested 1 g of ^13^C in leucine were (0.37 ± 0.25)% and (0.7 ± 1.5)%, respectively, and those for palmitic acid were (0.038 ± 0.030)% and (0.034 ± 0.070)%, respectively. Therefore, we concluded that the urine and faeces pathway could be omitted from the following experiment on carbon dynamics in our study. For glucose, we previously reported the negligible contribution of the urine and faeces pathways to the cumulative burden^21^.

### Metabolisms of ^13^C administered as various ^13^C -labelled compounds

We also administered the remaining target nutrient compounds,^13^C-labelled glutamic acid, glycine, phenylalanine, oleic acid, linoleic acid, and glucose to the volunteers who were healthy university students, three males and females each, and then we collected only breath and hair samples. All volunteers were administered each compound once daily for 4 successive days.

**Figure 4:**
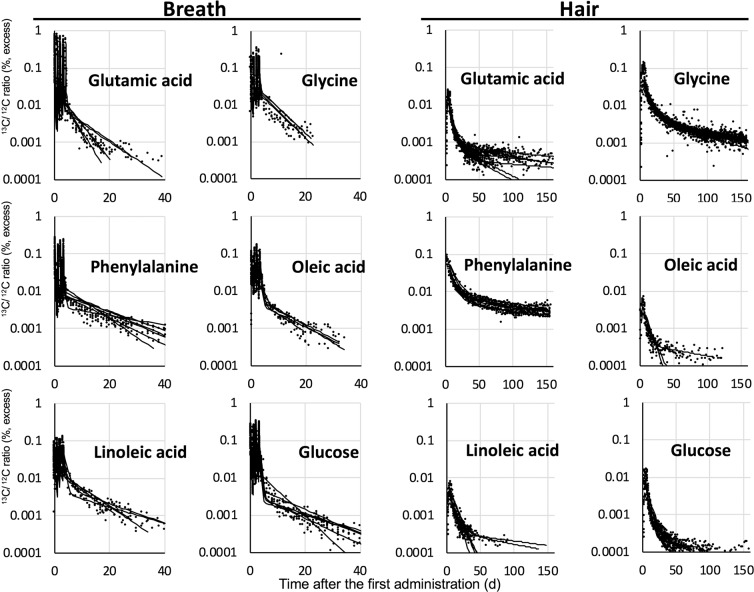
Ratio of ^13^C/^12^C in excreta (breath and hair) after oral administration of ^13^C-labelled compounds.

Figure 4 shows the ^13^C/^12^C ratio of the breath and hair samples after the first administration of each ^13^C-labelled compound. The ^13^C/^12^C ratios of hair samples decreased faster in the groups of volunteers administered oleic acid, linoleic acid, and glucose than in the other groups and reached the detection limit before the end of experiments. Consequently, we did not analyse parameters relating the slow carbon compartment in the other* pathway for some subjects. In contrast, the ^13^C/^12^C ratios of the groups of volunteers administered glycine and phenylalanine were kept relatively high throughout the experimental period, and were higher than those in the breath samples of the same volunteers toward the end of the experiment. These results reflected the higher utilization of carbon in amino acids for protein synthesis such as in hair growth than carbon in fatty acids and monosaccharides. The results of leucine and palmitic acid-administered group also showed a similar tendency (Fig. 1). However, amino acids in samples from volunteers in the glutamic acid-administered group decreased faster than other amino acids did. This observation is consistent with the reported high use of dietary glutamic acid in the small intestine^22^.

The metabolic model was also developed for each compound. The values of parameters of the breath and other* pathways were estimated and are summarized in Table 2. The parameters for subjects with only fast C compartment in the other* pathway were included in the summarization. Since the difference of results between sexes was not clear, the average values for individual data of both sexes were also shown in Table 2 and discussed below. The estimated distribution ratio of ^13^C for the breath pathway (*d*_*B*_) for leucine, glutamic acid, glycine, and phenylalanine were (68 ± 5)%, (84 ± 8)%, (80 ± 9)%, and (64 ± 7)%, respectively. Berlin and Tolbert^17^ reported an 86% recovery through the breath pathway after intravenous administration of glycine-[2-^14^C], which was consistent with the value of glycine in our experiment. Compared to glutamic acid and glycine, smaller proportions of leucine and phenylalanine were excreted through the breath pathway while a considerable portion was excreted through the other* pathway (*d*_*O*_, (30 ± 12)% and (35 ± 8)%, respectively). Leucine stimulates protein synthesis via the mammalian target of rapamycin complex 1 (mTORC1) mediating signalling pathway^23^ and, therefore, it might activate this pathway and consequently reduced its oxidation. However, such an effect has not been reported for phenylalanine. The distribution ratio of ^13^C of glucose in the breath pathway (*d*_*B*_, 90 ± 15%) was similar to the value reported in our previous paper (86 ± 10%)^21^.

**Table 2:** Parameters are explained in Fig. 2.

Parameter	Glutamic acid									Glycine								
	Male			Female			All			Male			Female			All		
	Mean	S.D.	n	Mean	S.D.	n	Mean	S.D.	n	Mean	S.D.	n	Mean	S.D.	n	Mean	S.D.	n
**(A) Estimated parameters of developed model for** ^**13**^**C in glutamic acid and glycine**.																		
*d*_*B*_	0.83	0.11	3	0.85	0.05	3	0.84	0.08	6	0.74	0.01	2	0.84	0.10	3	0.80	0.09	5
*d*_*Bf*_	0.75	0.05	3	0.72	0.01	3	0.73	0.03	6	0.37	0.05	2	0.36	0.03	3	0.36	0.03	5
*d*_*O*_	0.048	0.009	3	0.029	0.005	3	0.038	0.012	6	0.18	0.02	3	0.18	0.02	3	0.18	0.02	6
*d*_*Of*_	0.41	0.07	3	0.59	0.16	3	0.50	0.15	6	0.47	0.02	3	0.52	0.01	3	0.50	0.03	6
*k*_*B*_ (d^−1^)	42	16	3	56	32	3	49	24	6	37	14	2	25	9	3	30	12	5
*k*_*Bf*_ (d^−1^)	11	0	3	15	5	3	13	4	6	9.7	3.5	2	11	4	3	11	3	5
*k*_*Bs*_ (d^−1^)	0.27	0.05	3	0.15	0.06	3	0.21	0.08	6	0.16	0.00	2	0.18	0.02	3	0.17	0.02	5
*k*_*Of*_ (d^−1^)	0.21	0.01	3	0.17	0.02	3	0.19	0.03	6	0.11	0.02	3	0.098	0.003	3	0.10	0.01	6
*k*_*Os*_ (d^−1^)	0.0051	0.0014	3	0.016	0.011	3	0.010	0.009	6	0.0080	0.0006	3	0.0091	0.0030	3	0.0086	0.0021	6
*R*_*r*_	0.90	0.12	3	0.91	0.05	3	0.90	0.08	6	0.93	0.02	3	1.0	0.1	3	1.0	0.1	6
**Parameter**	**Phenylalanine**									**Oleic acid**								
	**Male**			**Female**			**All**			**Male**			**Female**			**All**		
	**Mean**	**S.D**.	**n**	**Mean**	**S.D**.	**n**	**Mean**	**S.D**.	**n**	**Mean**	**S.D**.	**n**	**Mean**	**S.D**.	**n**	**Mean**	**S.D**.	**n**
**(B) Estimated parameters of developed model for** ^**13**^**C in phenylalanine and oleic acid**.																		
*d*_*B*_	0.59	0.06	3	0.69	0.01	3	0.64	0.07	6	0.62	0.08	3	0.55	0.07	3	0.59	0.08	6
*d*_*Bf*_	0.37	0.04	3	0.41	0.04	3	0.39	0.05	6	0.53	0.24	3	0.66	0.05	3	0.59	0.17	6
*d*_*O*_	0.26	—	1	0.38	0.06	3	0.35	0.08	4	0.0067	—	1	0.012	0.006	2	0.010	0.005	3
*d*_*Of*_	0.35	—	1	0.30	0.03	3	0.31	0.04	4	—	—	0	0.37	—	1	0.37	—	1
*k*_*B*_ (d^−1^)	52	22	3	49	18	3	51	18	6	7.0	2.9	3	15	11	3	11	8	6
*k*_*Bf*_ (d^−1^)	9.1	0.24	3	8.3	4.2	3	8.7	2.7	6	4.6	1.8	3	1.9	0.5	3	3.2	1.9	6
*k*_*Bs*_ (d^−1^)	0.070	0.012	3	0.073	0.043	3	0.071	0.028	6	0.12	0.04	3	0.097	0.011	3	0.11	0.03	6
*k*_*Of*_ (d^−1^)	0.14	—	1	0.13	0.03	3	0.13	0.02	4	0.095	—	1	0.17	0.07	2	0.14	0.06	3
*k*_*Os*_ (d^−1^)	0.0061	—	1	0.0058	0.0013	3	0.0059	0.0011	4	—	—	0	0.0074	—	1	0.0074	—	1
*R*_*r*_	0.90	—	1	1.1	0.1	3	1.0	0.1	4	0.61	—	1	0.62	0.10	2	0.62	0.07	3
**Parameter**	**Linoleic acid**									**Glucose**								
	**Male**			**Female**			**All**			**Male**			**Female**			**All**		
	**Mean**	**S.D**.	**n**	**Mean**	**S.D**.	**n**	**Mean**	**S.D**.	**n**	**Mean**	**S.D**.	**n**	**Mean**	**S.D**.	**n**	**Mean**	**S.D**.	**n**
**(C) Estimated parameters of developed model for** ^**13**^**C in linoleic acid and glucose**.																		
*d*_*B*_	0.61	0.15	3	0.63	0.18	2	0.62	0.13	5	0.81	0.15	3	0.99	0.08	3	0.90	0.15	6
*d*_*Bf*_	0.48	0.17	3	0.43	0.23	2	0.46	0.17	5	0.71	0.14	3	0.85	0.00	3	0.78	0.11	6
*d*_*O*_	0.012	0.006	3	0.013	0.005	3	0.013	0.005	6	0.014	0.001	3	0.016	0.001	3	0.015	0.004	6
*d*_*Of*_	0.51	—	1	0.30	—	1	0.41	0.15	2	0.59	0.15	3	0.68	0.06	3	0.63	0.11	6
*k*_*B*_ (d^−1^)	21	10	3	16	4	2	19	8	5	43	14	3	69	7	3	56	17	6
*k*_*Bf*_ (d^−1^)	3.4	1.9	3	6.7	8.2	2	4.7	4.7	5	5.7	3.8	3	2.6	0.5	3	4.1	3.0	6
*k*_*Bs*_ (d^−1^)	0.095	0.039	3	0.085	0.034	2	0.091	0.033	5	0.094	0.055	3	0.064	0.014	3	0.079	0.049	6
*k*_*Of*_ (d^−1^)	0.097	0.023	3	0.14	0.05	3	0.12	0.04	6	0.14	0.01	3	0.30	0.10	3	0.22	0.11	6
*k*_*Os*_ (d^−1^)	0.0079	—	1	0.0056	—	1	0.0068	0.0016	2	0.0056	0.0037	3	0.022	0.014	3	0.014	0.013	6
*R*_*r*_	0.67	0.15	2	0.69	0.17	3	0.68	0.14	5	0.83	0.15	3	1.0	0.1	3	0.93	0.15	6

S.D., standard deviation.

The mean slow excretion rate constants (*k*_*Bs*_) of the breath pathway varied from 0.061 to 0.21 day^−1^ among the examined amino acids. These values were 5–20 times larger than the slow transfer coefficient of 0.0099 day^−1^ from the systemic tissue compartment to the carbon dioxide compartment in the generic model for workers recommended by the ICRP-134^5^. However, the slow excretion constants (*k*_*Os*_) of the other* pathway for the examined amino acids were from 0.0059 to 0.012 day^−1^ and comparable to the value of the slow compartment in the generic model for workers. Our result provides evidence to supports the existence of a carbon pool with a slow metabolic rate.

### Estimation of dose coefficient for members of public from ingested dietary ^14^C

As shown in Table 2B,C, the recovery ratios (*R*_*r*_) of the administered ^13^C in oleic and linoleic acids were markedly small at 0.62 and 0.68, respectively, while most of the administered ^13^C in the other examined compounds including palmitic acid were recovered in our experiment (Tables 1 and 2). Takeda *et al*.^9^ reported that the ^14^C concentration in adipose tissue in rats 100 days after administration of ^14^C-labelled oleic acid was three times higher than that of palmitic acid. Although palmitic acid is a main constituent of adipose tissue fatty acids in humans^24^, in this study, we assumed that the unrecoverable fraction of ^13^C in both unsaturated fatty acids and all nutrient compounds examined was stored in adipose tissue.

We further presumed that the carbon in adipose tissue had a mean residence time of 1.6 years, as estimated by Arner *et al*.^25^ for adipocyte lipids by analysing ^14^C originating from nuclear weapon tests. Since the ICRP does not provide the tissue weighting factor for adipose tissue, we also set two assumptions for this, 0 and 0.12 used for the remaining organs in the ICRP-103 guidelines^26^. The dose coefficients obtained with each assumption are called non conservative estimation (ER) and conservative estimation (EC) hereafter. The recovered fraction was assumed to be distributed uniformly in the whole-body carbon with a tissue weighting factor of unity. In considering the possible existence of additional carbon compartment, which was not detected in our study but have a longer half-life than that in the present slow carbon compartments, we adopted the EC as a moderate estimation. In addition to these estimations, we calculated the most conservative estimation (EX) adopting the tissue weighting factor of unity for unrecovered carbon. We solely adopted the value for the maximal estimation.

**Figure 5:**
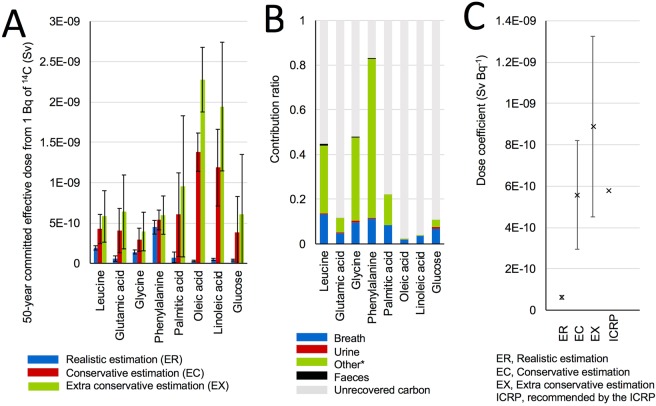
Dose estimation for ingested ^14^C. (**A**) Committed effective dose from 1 Bq of ^14^C in each compound. Error bars show standard deviation among subjects (n = 3–6). (**B**) contribution of ^13^C via each excretion pathway and unrecoverable carbon to the conservative committed dose (EC). (**C**) Dose coefficient for members of public from dietary ^14^C according to nutrition survey in Japan. Error bars show standard deviation.

Figure 5A shows the 50-year committed effective dose from 1 Bq of the ^14^C in labelled compounds after single ingestion calculated using each model developed. The ER values of the amino acids were generally higher than those of the fatty acids, whereas the EC and EX values were the opposite. Glucose showed similar ER values to those of the unsaturated fatty acids, whereas the EC and EX values are similar to those of amino acids. The assumption about the unrecoverable fraction played an extremely important role in our estimation of the dose because of the assumed long half-life of unrecovered carbon. The relative contribution of each pathway including the unrecoverable fraction to the EC is shown in Fig. 5B. It is noteworthy that parameters of the urine and faeces pathway in the first experiment were used for the other compounds (details are shown below), and the contribution of these pathways was very minor (<0.8%). As shown in Fig. 5B, the unrecoverable fraction contributed significantly to the committed dose when we adopted a tissue weighting factor of 0.12 to adipose tissue.

We estimated the dose coefficient for members of public based on ingestion of a Japanese diet^27^, which was uniformly labelled by ^14^C and had the nutritional composition shown in Table 3. The nutrition in the diet was classified into groups which was represented by each single compound examined in our study. The proportion of carbon ingested as the nutrition groups to total carbon ingestion rate was obtained by using literature data^28,29^ (details are shown below). The total daily ingestion of 300 g carbon per day of ICRP reference man was divided by the proportion of carbon ingestion rate each and inputted to each single compound model. The dose coefficient was obtained by summing up the 50-year committed effective dose from each representative compound comprising the diet with a total ingestion of 1 Bq ^14^C.

**Table 3:** Nutritional composition in Japanese diet and corresponding models used to calculate dose coefficient.

	Nutrition								
	Proteins				Lipids			Carbohydrates	Sum
					SFA	MU	PU		
Carbon ingestion* (g-dry d^−1^)	71.8				17.6	22.6	20.8	254.0	386.8
	BCAA	Glycine	AAA	Others					
Proportion of amino acid group in protein ingestion rate** (%)	18.2	4.3	12.7	64.8					
Carbon concentration^†^ (%)	54	32	60	53	75	77	77	44	
Carbon ingestion rate (g d^−1^)	7.1	1.0	5.5	24.7	13.2	17.4	16.0	111.8	196.6
Proportion to total carbon ingestion rate	0.036	0.0050	0.028	0.13	0.067	0.089	0.082	0.57	1.0
Represented model	Leucine	Glycine	Phenyl-alanine	Glutamic acid	Palmitic acid	Oleic acid	Linoleic acid	Glucose	

*, National Health and Nutritional Survey by the Ministry of Health, Labour and Welfare, Japan^27^; **, proportion of each amino acid group intake to protein intake^28^; ^†^^29^.

SFA, saturated fatty acid; MU, mono-unsaturated fatty acid; PU, poly-unsaturated fatty acid and others.

BCAA, branched chain amino acid (leucine, isoleucine, and valine); AAA, aromatic amino acid (phenylalanine, histidine, and tryptophan).

The ER of the Japanese diet was estimated to be (6.2 ± 0.9) × 10^−11^ Sv Bq^−1^ (Fig. 5C), which was nine and two times smaller than the current dose coefficient of 5.8 × 10^−10^ Sv Bq^−1^ for members of public as recommended by the ICRP-72^7^ and 1.6 ×10^−10^ Sv Bq^−1^ for workers adopting the generic multi-compartment model by ICRP-134^5^, respectively. The EC and EX were (5.6 ± 2.6) × 10^−10^ and (8.9 ± 4.4) × 10^−10^ Sv Bq^−1^, respectively (Fig. 5C). These conservative values were not significantly different from the current dose coefficient for members of public^7^.

As described before, there is no data about metabolism of carbon in a broad range of nutrients administered orally, this work firstly provides the experimental base of dose coefficient for members of public. Since the unrecoverable fraction was very important for the estimation of the dose coefficient in our research, further studies are necessary to clarify the metabolism of ^14^C in various nutrients.

## Materials and Methods

### Administration of ^13^C-labelled compounds

In the first experiment to select the major excretion routes for carbon in amino and fatty acids, an aliquot of uniformly ^13^C-labelled leucine and palmitic acid (Cambridge Isotopes, Inc., Tewksbury) was administered to healthy adult Japanese male sedentary workers aged 41 ± 9 and 40 ± 5 years old (n =3 each), respectively. The dose was 1.0 g ^13^C per person. We followed the administration procedure described in a previous paper^21^. Briefly, the ^13^C-labelled compounds were orally ingested by each volunteer at 12:15 immediately before eating lunch on experimental day 0. An additional 0.1 g of ^13^C in the compounds was similarly administered on day 112 after the initial administration to mark that day in the hair samples (see below). To avoid background fluctuations in the ^13^C/^12^C ratio by over-eating of ^13^C-rich foods, nutritionally balanced foods were provided to the volunteers throughout the experimental period from day −7 to 112, although they were able to eat other foods under certain circumstances.

In the second experiment using various ^13^C-labelled compounds, uniformly ^13^C-labelled glutamic acid, glycine, phenylalanine, oleic acid, linoleic acid, and glucose (Cambridge Isotopes, Inc., Tewksbury) were administered to healthy adult Japanese male and female volunteers (n = 3, each) aged 25 ± 7 years once a day at 12:05 on 4 successive days. The doses of those compounds were 8.9, 7.1, 11.9, 11.0, 16.4, and 35.6 mg ^13^C kg^−1^ day^−1^, respectively. Additional doses of ^13^C-labelled compounds were administered on day 160 for marking the ^13^C/^12^C ratio of hair samples and the volunteers freely ate meals without calorific restrictions.

### Sample collection and determination of ^13^C/^12^C ratio

In the first experiment, breath, urine, faeces, and hair samples were periodically collected and the ^13^C/^12^C ratio was analysed following the procedure described in a previous paper^21^. Briefly, breath and urine samples were collected daily when the volunteers rose in the morning from day −7 to 112. Faecal samples were collected once before day 0 and at every defecation from day 0 to 14. On day 0, additional breath and urine samples were collected frequently after administration. Five hair samples from each subject were collected on day 119 and used as representative samples of the other* pathway. Then, 10 cm of each hair starting from the root was cut into 1-mm sections and the ^13^C/^12^C ratio determination of each showed two peaks in the ^13^C/^12^C ratio corresponding to the first and second administration of ^13^C-compounds. According to the section number of the two peaks, the experimental date for each section between the two peaks was determined under an assumption of uniform hair growth. In the second experiment, breath samples were collected until day 160 and hair samples were obtained by brushing on day 167.

The ^13^C/^12^C ratio was measured using a mass spectrometer (Delta V Advantage; Thermo Electron, Waltham, MA, USA) coupled with an elemental analyser (FlashEA 1112NC; Thermo Electron) and a gas chromatograph (MAT GC; Thermo Electron) for combusting organic samples and purifying CO_2_. Breath samples were collected by the volunteers themselves, and some samples showed clearly inconceivable results such as similar ^13^C/^12^C ratios over considerable periods of breath sample collection, which may have been caused by collecting the samples on a single day instead of once daily sampling on sequential days. The whole data of such individuals were discarded, which decreased the number of samples from three in Table 2. Some volunteers also dropped out of the sampling procedure for various reasons.

### Constructing multi-compartment metabolic model by regression analysis

The ^13^C distribution ratios and excretion rate constants of all compartments in the four excretion pathways for leucine and palmitic acid were determined using the least squares fitting method with the statistical analysis software R version 2.15.2., while only parameters for the breath and other* pathway were determined for the other six examined compounds. The detailed procedures used were described in a previous published paper^21^. When urine and hair samples had no increase phase, the values of *k*_*U*_ and *k*_*O*_ were fixed to those of *k*_*B*_ obtained from the same volunteers in the regression analysis.

### Estimation of dose coefficient for members of public from dietary ^14^C

The 50-year cumulative body burden of ^14^C after a single ingestion of 1 Bq of ^14^C in each compound (*B*_*C*_ Bq d) was calculated using the metabolic model and its parameters described above, under the assumption of the same metabolic behaviour of ^13^C and ^14^C. The parameters of the urine and faeces pathway for leucine and palmitic acid were used for the other amino acids and fatty acids. For glucose, the parameters of the urine and faeces pathways reported in our previous paper^21^ were adopted. The total cumulative burden was derived as a sum of burdens from the recoverable and unrecoverable fractions with designated proportions *R*_*r*_ and 1−*R*_*r*_, respectively. For the recoverable fraction, the burden was calculated using following equation:

2 $${B}_{C}\approx \sum _{m}\frac{{d}_{m}}{{k}_{m}}+\sum _{m}\sum _{n}\frac{{d}_{m}{d}_{mn}}{{k}_{mn}}$$

where, every *d*_*m*_ was divided by *R*_*r*_ when *R*_*r*_ was larger than unity.

The 50-year committed effective dose (Sv) from ingestion of 1 Bq ^14^C was calculated from the 50-year cumulative burden by using the ratio of absorbed dose to ^14^C radioactivity, radiation weighting factor of beta-ray from ^14^C (1.0) and tissue weighting factors. As previously described, for the unrecoverable fraction, we used a mean residence time of 1.6 years (excretion rate constant of 0.0017 d^−1^)^25^ and tissue weighting factors of 0, 0.12, and unity for the estimation of ER, EC, and EX, respectively.

The Japanese typical nutrition intake rates were cited from the annual nationwide National Health and Nutrition Survey conducted by the Ministry of Health, Labour and Welfare, Japan^27^. In addition, we used the proportion of each amino acid as reported by Iwaya^28^ to obtain the ingestion rate of each amino acid group including branched chain amino acid (BCAA), major amino acid, glycine, and aromatic amino acid (AAA) groups (Table 3). We assumed that the metabolism of each amino acid group was the same as that of the represented compound examined in our study as shown in Table 3. The lipids and carbohydrates were also represented by each single compound. Then, the ingestion rate of carbon in each nutrition group was calculated according to the weight percentage of carbon in the representative compound (Table 3)^29^, followed by obtaining the proportion of carbon ingestion rate of each group to the total carbon ingestion rate. Then, the input to each compound model was calculated from the total carbon ingestion rate of 300 g carbon per day of ICRP reference man^6^ and the proportion of carbon ingestion rate. Finally, the dose coefficient for members of public from dietary ^14^C was calculated by summing up the committed effective doses of ^14^C and weighting proportion of ^14^C in each compound up to 1 Bq ^14^C in the diet.

### Ethical considerations

The study protocol conformed to the ethical guidelines of the 1975 Declaration of Helsinki as reflected in the prior approval of the Institute for Environmental Sciences Review Board for Human Subject Experiments. Written informed consent was obtained from each volunteer prior to participation.

## Supplementary information

Supplementary information.

## Data Availability

The datasets generated and analysed during the current study are available from the corresponding author on a reasonable request.

## References

[1] Koarashi J, Akiyama K, Asano T, Kobayashi H (2005). Chemical composition of ^14^C in airbone release from the Tokai reprocessing plant, Japan. Radiat. Prot. Dosimetry..

[2] MaCartney M, Baxtre MS, Scott EM (1988). Carbon-14 discharged from the nuclear fuel cycle: 2 Local effects. J. Environ. Radioact..

[3] Veluri VR, Boone FW, Palms JM (1976). The environmental impact of ^14^C released by a nuclear funnel-reprocessing plant. Nucl. Safety..

[4] International Commission on Radiological Protection (ICRP), Limits for intakes of radionuclides by workers. ICRP Publication 30. Ann. ICRP 2 (Elsevier (1979).

[5] International Commission on Radiological Protection (ICRP), Occupational intakes of radionuclides: Part 1. ICRP Publication 134. Ann. ICRP 45 (Elsevier (2016).

[6] International Commission on Radiological Protection (ICRP), Report on the task group on reference man. ICRP Publication 23. Ann. ICRP (Elsevier (1975).

[7] International Commission on Radiological Protection (ICRP), Age-dependent doses to the members of the public from intake of radionuclides: Part 5 Compilation of ingestion and inhalation coefficients. ICRP Publication 72. Ann. ICRP 26 (Elsevier 1995).

[8] Paquet F, Bailey MR, Leggett R (2016). Assessment and interpretation of internal doses: uncertainty and variability. Ann. ICRP..

[9] Takeda H, Fuma S, Miyamoto K, Nishimura Y, Inaba J (1998). Biokinetics and dose estimation of radiocarbon in rats. Radiat. Prot. Dosimetry..

[10] Takeda H (2009). Biokinetics of radiocarbon ingested as a food in rats. Health Phys..

[11] Takeda H (2010). Comparative biokinetics of radiocarbon ingested as compounds or foods in rats. Health Phys..

[12] Crout NM, Mayes RW, Beresford NA, Lamb CS, Howard BJ (1998). A metabolic approach to simulating the dynamics of C-14, H-3 and S-35 in sheep tissues. Radiat. Environ. Biophys..

[13] Lappin G, Garner RC (2004). Current perspective of ^14^C-isotope measurement in biomedical accelerator mass spectrometry. Anal. Bioanal. Chem..

[14] Gunnarsson M (2007). Long term biokinetics and radiation exposure of patients undergoing ^14^C-glycocholic acid and ^14^C-xylose breath tests. Cancer Biother. Radiopharm..

[15] Malmendier CL, Delcroix C, Berman M (1974). Interrelation in the oxidative metabolism of free fatty acids, glucose, and glycerol in normal and hyperlipemic patients. J. Clin. Invest..

[16] Simmons PS, Nissen S, Haymond MW (1982). Total body absorbed radiation dose from 1-^14^C-alanine for human studies. Health Phys..

[17] Berlin NI, Tolbert BM (1953). Metabolism of glycine-2-^14^C in man: V. further consideration of pulmonary excretion of ^14^CO_2_. Proc. Soc. Exp. Biol. Med..

[18] Stenström K (1996). Application of accelerator mass spectrometry (AMS) for high-sensitivity measurements of ^14^CO_2_ in long term studies of fat metabolism. Appl. Radiat. Isot..

[19] Gunnarsson M (2003). Biokinetics and radiation dosimetry for patients undergoing a glycerol tri[1-^14^C]oleate fat malabsorption breath test. Appl. Radiat. Isot..

[20] Stenström K, Unkel I, Nilsson CM, Raaf C, Mattsson S (2010). The use of hair as an indicator of occupational ^14^C contamination. Radiat. Environ. Biophys..

[21] Masuda T (2016). Biokinetics of 13C in the human body after oral administration of ^13^C-labeled glucose as an index for the biokinetics of ^14^C. J. Radiol. Prot..

[22] Reeds PJ, Burrin DG, Davis TA, Stroll B (1998). Amino acid metabolism and the energetics of growth. Arch. Tieremahr..

[23] Dickinson JM (2011). Mammalian target of rapamycin complex 1 activation is required for the stimulation of human skeletal muscle protein synthesis by essential amino acids. J. Nutr.

[24] Insull W, Lang PD, His BP, Yoshimura S (1969). Studies of arteriosclerosis in Japanese and American men. J. Clin. Invest..

[25] Arner P (2011). Dynamics of human adipose lipid turnover in health and metabolic disease. Nature.

[26] International Commission on Radiological Protection (ICRP). The 2007 recommendations of the International Commission on Radiological Protection. ICRP Publication 103. Ann. ICRP 37 (Elsevier (1997). 10.1016/j.icrp.2007.10.003 18082557

[27] Ministry of Health, Labour and Welfare, Japan. The National Health and Nutrition Survey, Japan [(accessed on 12th March 2020)]; Available online: https://www.mhlw.go.jp/bunya/kenkou/kenkou_eiyou_chousa.html. (In Japanese).

[28] Iwaya M (1984). Amino acid composition of dietary protein ingested by the Japanese and its nutritional evaluation. Jpn. J. Nutr. Diet..

[29] Nakamura M, Yuyama Y (2005). Development of a composition database for various types of biomass. Tech. Report NIRE.

