# Supplementary material for: Estimation of dietary 14C dose coefficient using 13C-labelled compound administration analysis
Source: Sci Rep. 2020 May 18;10:8156. doi: 10.1038/s41598-020-64954-w (PMC7235250; doi:10.1038/s41598-020-64954-w)
Supplement: Supplementary file 1 — Supplementary information. [file 41598_2020_64954_MOESM1_ESM.doc]

**Supplementary Information**

**Estimation of** **dietary ^14^C dose coefficient using ^13^C-labelled compound administration** **analysis**

Tsuyoshi Masuda^1*^, Toshitada Yoshioka^2^, Tomoyuki Takahashi^3^, Hiroshi Takeda^4^, Hideo Hatta^5^, Kensaku Matsushita^1^, Yasuhiro Tako^1^, Yuichi Takaku^1^, and Shun'ichi Hisamatsu^1^

^1^ Institute for Environmental Sciences, Aomori, Japan

^2^ Hirosaki Gakuin University, Aomori, Japan

^3^ Kyoto University, Osaka, Japan

^4^ National Institute of Radiological Sciences, Chiba, Japan

^5^ University of Tokyo, Tokyo, Japan

***Correspondence to masuda@ies.or.jp**


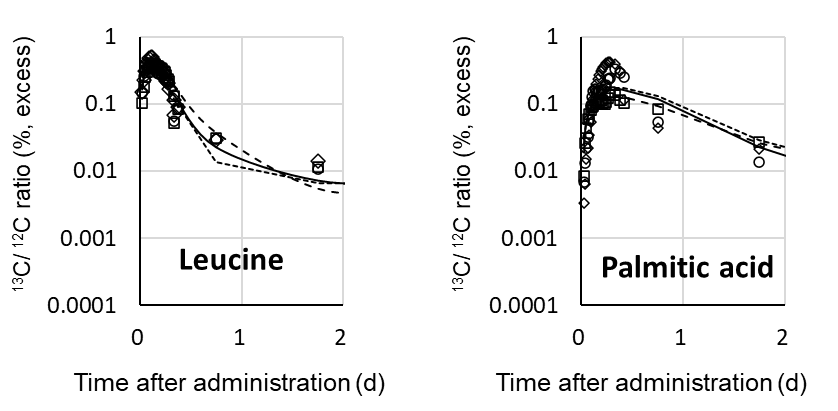


Figure S1. Ratio of ^13^C/^12^C in breath after oral administration of ^13^C-labeled leucine and palmitic acid shown in Figure 1. The first two day after administration are shown in order to better illustrate initial behavior. Left and right panel are corresponding to the upper left and lower left panel of Figure 1, respectively. Circles, diamonds, and squares represent volunteer 1, 2, and 3, respectively. Lines were fitted using model developed in this study.
